# Nonverbal Synchrony in Social Interactions of Patients with Schizophrenia Indicates Socio-Communicative Deficits

**DOI:** 10.1371/journal.pone.0145882

**Published:** 2015-12-30

**Authors:** Zeno Kupper, Fabian Ramseyer, Holger Hoffmann, Wolfgang Tschacher

**Affiliations:** 1 Division of Molecular Psychiatry, Translational Research Center, University Hospital of Psychiatry, University of Bern, Bern, Switzerland; 2 Department for Clinical Psychology and Psychotherapy, University of Bern, Bern, Switzerland; 3 ARTORG—Gerontechnology and Rehabilitation, University Hospital of Old Age Psychiatry, University of Bern, Bern, Switzerland; 4 Center for Psychiatric Rehabilitation, University Hospital of Psychiatry, University of Bern, Bern, Switzerland; 5 Division of Systems Neuroscience of Psychopathology, Translational Research Center, University Hospital of Psychiatry, University of Bern, Bern, Switzerland; Maastricht University, NETHERLANDS

## Abstract

**Background:**

Disordered interpersonal communication can be a serious problem in schizophrenia. Recent advances in computer-based measures allow reliable and objective quantification of nonverbal behavior. Research using these novel measures has shown that objective amounts of body and head movement in patients with schizophrenia during social interactions are closely related to the symptom profiles of these patients. In addition to and above mere amounts of movement, the degree of synchrony, or imitation, between patients and normal interactants may be indicative of core deficits underlying various problems in domains related to interpersonal communication, such as symptoms, social competence, and social functioning.

**Methods:**

Nonverbal synchrony was assessed objectively using Motion Energy Analysis (MEA) in 378 brief, videotaped role-play scenes involving 27 stabilized outpatients diagnosed with paranoid-type schizophrenia.

**Results:**

Low nonverbal synchrony was indicative of symptoms, low social competence, impaired social functioning, and low self-evaluation of competence. These relationships remained largely significant when correcting for the amounts of patients‘ movement. When patients showed reduced imitation of their interactants’ movements, negative symptoms were likely to be prominent. Conversely, positive symptoms were more prominent in patients when their interaction partners’ imitation of their movements was reduced.

**Conclusions:**

Nonverbal synchrony can be an objective and sensitive indicator of the severity of patients’ problems. Furthermore, quantitative analysis of nonverbal synchrony may provide novel insights into specific relationships between symptoms, cognition, and core communicative problems in schizophrenia.

## Introduction

Nonverbal behavior is crucial in human communication, given its phylogenetic and ontogenetic primacy [1,2]. Common understanding, empathy, and agreement are largely expressed nonverbally via interpersonal adaptation [3] and coordination. This interactional synchrony, the coordination between two interactants’ movements, based on imitation of dynamic aspects of nonverbal behavior, was first described by Condon and Ogston [4] with regard to its importance in normal and pathological behavior patterns. Its importance is evident early in life [5] and a large body of research has confirmed the importance of imitation in social interactions and identified various psychological moderators of imitation [6,7]. In accordance with this research, the term imitation (or "behavioral mimicry") should not be restricted to deliberate behavior. Rather, the spontaneous, automatic imitation of not only gestures, postures, and mannerisms but also the dynamics of movement, has to be considered pervasive in human interaction [6]. Imitation has a neuroanatomical correlate in the brain. Mirror neurons, first discovered in macaque primates [8,9], have inspired numerous studies on imitation in nonhuman primates and humans [10,11]. This has ultimately resulted in a body of evidence regarding humans, associating neurobiological structures with empathy, theory of mind, autism, and schizophrenia [12,13]. The study of movement in schizophrenia can open new avenues for translational research [14–18].

Nonverbal synchrony may be markedly reduced in interactions involving persons with schizophrenia, in which impaired communication and social functioning are prominent problems [19–23]. Communication problems in schizophrenia have been examined via approaches focusing on social cognition [24], including neurophysiological models aimed at clarifying the putative interactions between positive and negative symptoms, social cognition, and brain mechanisms [25]. On a clinical level, psychotic symptoms, such as pronounced thought disorders, can have obvious disturbing effects on verbal and nonverbal behavior. However, there are more subtle alterations in communication, which have been known to experienced practitioners for some time [26]. The subtle, yet obvious, lack of synchrony in verbal and nonverbal interactive behaviors may be the key to clinicians’ intuitive experience of a "praecox feeling" [27] owing to the presence of schizophrenia in patients. In addition, these communicative problems appear related to the "clinical core of schizophrenia" in terms of the European tradition of psychiatry [28]. In this view, psychotic symptoms themselves were seen as secondary and state-related, whereas expressions of fundamental "core" disorders were seen as traits. These core disorders were described using various terms, particularly "schizophrenic autism" [29]. Autistic traits are described in many clinical manifestations, including a lack of communication and attunement to patients’ social environments. Many of the features previously seen as being part of the clinical core of schizophrenia are no longer part of the present operational definitions of schizophrenia in the DSM-5 or ICD-10 [28]. Consequently, lack of synchrony in social interaction may not only be an important feature of social functioning in schizophrenia but could also provide a clinically accessible indicator of a possible key feature of the disorder.

Many studies have found indicators of persistent problems of nonverbal communication in patients with schizophrenia, such as reduced facial expression [30–33]. Their deficits in social skills have been analyzed using observer-based rating scales [17,34], and more recently, with objective measures of movement [21,22,35–38]. Nonverbal behavior in patients with schizophrenia can be assessed objectively using ordinary video recordings. The objectively assessed amount of movement observed in patients with schizophrenia during social interaction can be closely related to the symptom profiles of these patients [35]. A recent review [37] found a reduction in patients’ nonverbal behaviors, specifically those related to inviting interaction with others, which was associated with poorer social adjustment. This reduction was most prominent in patients with negative symptoms. However, positive symptoms were associated with an increase in nonverbal behaviors.

Due to lacking technology, the symptoms and behavioral signs of schizophrenia were commonly assessed via clinical judgment or expert ratings [18,37]. Developments in high-end multimedia equipment, together with advances in computing speed and analysis strategies, have facilitated computer-based, automated quantification of movement and the creation of novel methods for analyzing nonverbal communication. One such innovative method is Motion Energy Analysis (MEA) [35][39–42]. MEA utilizes digitized movies and is based on frame-differencing algorithms [43–46]. Successive frames (pictures) of a digitized movie are analyzed to determine the degree of change from one frame to the next in predefined regions of interest. In psychological research, frame-differencing methods were initially implemented in the analysis of courtship behavior [44], and MEA has since been adapted to analyze psychotherapy sessions [39–41] and student interactions [47,48]. We previously applied MEA to the videotaped role-play interactions of 27 stabilized outpatients with paranoid schizophrenia [35]. Movement parameters (percentage of time in movement and movement speed) were correlated with symptom ratings from independent PANSS interviews and proved to be highly reliable. Reduced movement and movement speed correlated with negative symptoms. Patients with negative symptoms differed from normal interactants by showing significantly reduced head and body movement. A close and theoretically meaningful association was found between the objective movement parameters and symptom profiles. In this previous report, only measures of patients' movement were considered.

Studies using objective quantitative measurements of nonverbal behavior in patients with schizophrenia are still scarce [37]. Very few have quantitatively examined nonverbal synchrony in social interaction. Many studies utilized ethological coding for the assessment of nonverbal coordination [49–52], while more recent approaches relied on computer-based measures [36]. Lavelle examined interpersonal coordination in a triadic setting involving one patient with schizophrenia and two healthy interactants; results showed an initially lower coordination between patients and healthy participants, and lower nonverbal synchrony was associated with lower rapport [53]. A number of studies have evaluated more laboratory-based forms of intentional and unintentional motor-coordination using e.g. hand-held pendulums operated by schizophrenia patients or unaffected relatives and control participants [22,54].

Our main goal was to explore the importance of nonverbal synchrony in schizophrenia, using quantitative measurement technology. This report is based on video recordings involving 27 patients with paranoid schizophrenia mentioned above [35]. In our previous report, the analysis was restricted to the amount of patients’ movement (percentage of movement above an objective threshold); in the current study, we analyzed the movement time-series of both patients and interactants to calculate each dyad's nonverbal synchrony. We hypothesized that the degree of nonverbal synchrony between patients and interactants is associated with patients’ symptom factor scores, cognitive variables, and social functioning. We additionally explored the specific relationships between nonverbal synchrony and individual symptoms. To examine the specificity of these relationships, we tested the association of nonverbal synchrony with symptoms, cognitive variables, and social functioning while correcting for the raw amount of patients’ movement.

## Methods

### Participants

In the present study, nonverbal synchrony was assessed objectively using MEA in 378 brief, videotaped role-play scenes involving 27 stabilized outpatients diagnosed with paranoid-type schizophrenia. Seven participants were female. All patients were clinically stabilized, and none suffered from further severe medical or neurological disease. Patients generally showed moderate to low levels of psychopathology, as assessed on the Positive and Negative Syndrome Scale (PANSS) [55]. Patients' mean score on the Global Assessment of Functioning scale (GAF, Axis V in DSM-IV) was 44.7 (*SD* = 4.3), indicating that they were seriously impaired in global levels of personal and social functioning. All patients provided written informed consent to participate in the research procedures. The assessment procedures are described below.

### Measures

PANSS factors, according to the model of Lindenmayer et al. [56], were used and included five symptom factors: positive, excitement, cognitive, negative, and depression. Role-play tests (RPT) are psychometrically sound and valid instruments to measure social competence in schizophrenia [57]. The particular RPT used in this study [58] was the German adaptation of an RPT developed by Bellack et al. [59]. It consisted of 14 social scenes representing three response domains: initiating conversations, resisting unfair treatment, and expressing appreciation. Staff members working at a rehabilitation unit and research assistants served as interactants in the role-play interactions. Problems with specific domains of social functioning were assessed using the Disability Assessment Schedule—Mannheim version (DAS-M) [60]. Cognitive measures included the German version of the Wechsler Adult Intelligence Scale (WAIS) [61], the Frankfurt Self-Concept Scales (FSKN) [62], and the Coping with Stress Questionnaire (SVF) [63].

### Video-Based Quantification of Nonverbal Behavior

**Fig 1:**
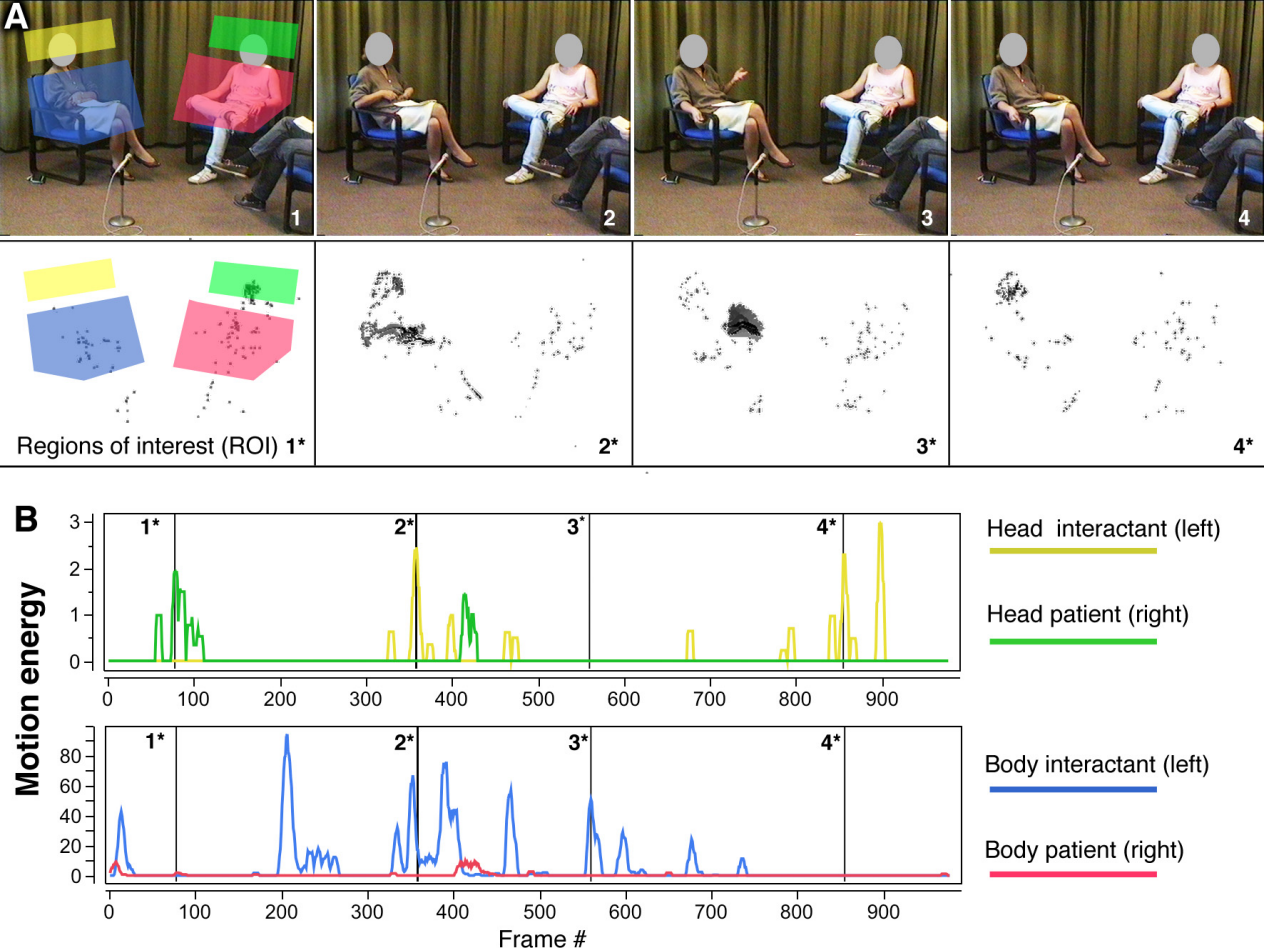
Video-based quantification of nonverbal behavior with motion energy analysis (MEA)—Regions of interest (A) and motion energy time series (B).

Role-play scenes were recorded in VHS format using a fixed camera. Individual role-play scenes had a median duration of 52 seconds. A total of 378 movie sequences resulted from recordings of 27 patients in 14 scenes of the RPT. Digital conversion of VHS tapes was conducted at 15 frames per second with an analogue-to-digital converter. MEA [41,42] is based on a frame-differencing algorithm, which quantifies the amount of change from one movie frame to the next. Given the fixed camera position and lack of movement within the range of the camera, there is no difference between consecutive frames. However, when a person or an object moves, motion energy may be defined as the amount of change observed from one frame to the next. This kind of objective quantification may be performed on the entire image or on selected parts thereof. Predefined regions of interest (ROI), for example, a region including a seated person's head, may be chosen freely. For the assessment of patient movement in role-play scenes, two ROIs were defined. They pertained to the patient's head (without the neck) and the upper body (from the base of the chair up to the shoulders), as seen in Fig 1.

Fig 1 depicts a role-play interaction and exemplifies the measurement of head and body movement in the present study. Panel A, pictures 1 to 4, includes still pictures from one role-play interaction. Picture 1 provides definitions for the head and body ROIs. The patient’s ROIs are shown in green and red, and the interactants' respective ROIs are colored yellow and blue. Below each picture (1* to 4*), areas of detected movement are shown. In all four ROIs, changes in grayscale values were detected and recorded separately as numerical streams of data, generating four continuous time-series measuring the amount of movement in the head and body regions, as shown in panel B. The measurement procedures were repeated for each subject in all 378 role-play scenes. In our previous report [35], we were able to establish the reliability of the measurements and reported that movement was not negatively related to neuroleptic medication or the intake of illicit drugs, particularly marihuana. We restricted our subsequent analysis to the head regions, because previous research has shown these regions to be related more closely to symptom profiles [35].

### Quantification of Nonverbal Synchrony

The quantification of movement synchrony corresponded to the procedures outlined by Ramseyer et al. [41]. We quantified nonverbal synchrony for all 378 role-play interactions (14 role-play interactions for each of the 27 patients). The term "head synchrony" is used in the remainder of this article to indicate our measure of movement synchrony between patients’ and interactants’ head movements. The time-series of motion energy for both patient and interactant were cross-correlated [64,65] in consecutive windows corresponding to the length of each of the 14 interactions. Cross-correlations for positive and negative time-lags up to 5 s in steps of 0.067 s were computed for each interaction-window by step-wise shifting one time-series in relation to the other (75 steps, corresponding to 5 s, in each direction of positive and negative lags). Cross-correlations were then standardized (Fisher’s Z) and their absolute values were aggregated over each of the 14 role-play interactions. Each role-play dyad was therefore characterized by a synchrony score, which was indicative of the average level of coordination between patients' and interactants' head movements during the role-play test. The median value for head synchrony across all 14 role-play interactions, his or her global synchrony score, was used for each patient. Median synchrony scores, rather than mean values, were used to prevent a possible bias resulting from erratic scores in interactions with no or very few movement in either of the two interactants. Nevertheless, median and mean synchrony scores were highly correlated (*r* = 0.86, *n* = 27). For the resulting median global synchrony measure, skewness (*v* = 0.254) and kurtosis (*w* = 0.347) did not suggest a departure from a normal distribution.

In addition to this global synchrony measure, we were interested in the direction of synchrony–targeting the question who of the interactants moves first and therefore appears to set the timing for the interaction partner [66,67]. This facet of nonverbal coordination has previously been called "pacing" and "leading" [41] because it takes into account whether a specific interactant's behavior occurred first (= leading / the person is being imitated), or whether this interactant imitated the other person (= pacing / imitating). Using the same time-lagged cross-correlations described above, we chose the patient as the point of reference for this distinction, and identified how much the patient imitated the interactant (imitation) by a lag of up to –5 s and how much the patient was imitated by the interactant (being imitated) by a lag of up to +5 s. Therefore, nonverbal synchrony was calculated as I) a global value, comprising all available cross-correlations of ±5 seconds (*N* = 151 cross-correlations, including the lag of zero) and II) a subscore indicating how much each patient imitated the interaction partner (*n* = 75 cross-correlations), and III) a subscore indicating how much the patient was imitated by the interaction partner (*n* = 75 cross-correlations).

### Control for spurious correlations: effect-size of nonverbal synchrony

A long-standing criticism of previous work on nonverbal synchrony was the lack of a statistical control for so-called pseudosynchrony–synchrony that is caused by random coincidence [68]. In line with previous solutions to this problem [69,70], we generated surrogate datasets (*N* = 100) by segment-wise shuffling of the original data, so that all movement segments of the patient were dislocated randomly with respect to segments of the interactant. Pseudosynchrony–the synchrony obtained in shuffled datasets–was calculated identically to the genuine synchrony of the original data. For the comparison of nonverbal synchrony versus pseudosynchrony, effect sizes (Cohen's *d*), based on the mean value of 100 shuffled surrogate data, were computed [70]. The pseudointeractions generated by this bootstrap procedure thus provided a base-level of pseudosynchrony, with which the genuine synchrony of interactions could be compared. Such a contrast yielded an estimate of the size of the synchrony effect, and it also allowed the comparison of effect-sizes across different interaction settings and dyads.

### Data Analysis

Descriptive data for all measures are given in Table 1. For the main analysis, median scores for head synchrony for each patient were correlated with PANSS factors, individual PANSS symptoms, social skills ratings for the role-play scenes, and cognitive variables (Pearson’s correlation). An additional correlation analysis, partialing out the influence of the amount of movement, was used to test the independent contributions of head synchrony in predicting negative symptoms. Finally, correlations were calculated for the two synchrony subscores that indicated the extent to which the patients imitated and were imitated by their interaction partners. As indicated above, the main goal of this study was to explore the relationships of synchrony with symptom factors. Adjusted significance levels based on false decovery rate (FDR) control procedures [71,72] were provided for the main analysis, relating synchrony with symptom factors. Additionally, detailed results including correlations with single symptom scores and subscales of social measures were provided. Adjusted signifance levels were also reported for relationships between synchrony and these variables. However, due to the small sample size, these results and all correlations between variations of the head movement parameter (percentage of movement, residual synchrony correcting for movement, sychrony based on patient imitating vs. sychrony based on patient being imitated) are exploratory. These results are reported to provide directions for further research. SAS (Version 9.3) and JMP statistical software (Version 10, SAS Institute, Cary, NC) were used for the statistical analysis.

**Table 1:** Descriptive data for nonverbal synchrony, symptoms, cognitive, and social measures (n = 27).

Measure	Mean	Median	SD
*Head movement parameters (MEA) / synchrony*			
Percentage of time moving	20.0	17.6	10.24
Synchrony–patient imitates	0.068	0.066	0.015
Synchrony—patient is imitated	0.065	0.065	0.016
Overall head synchrony	0.070	0.071	0.015
*Symptoms (PANSS)* ^a^			
Positive factor	12.7	13	3.62
Negative factor	14.3	13	5.84
General factor	27.9	27	7.42
PANSS total	55.0	52	14.76
*Social competence (role play test)* ^b^			
Global score	2.69	2.75	0.69
Gaze / eye contact	1.45	1.17	0.62
Affect	1.49	1.33	0.47
Speech duration	1.47	1.5	0.36
Meshing/latency	1.38	1.33	0.27
*Social impairment (DAS-M)*			
Self-care and personal presentation (DAS-M11, n = 21)	1.10	1	1.14
Leisure activities (DAS-M12, n = 21)	2.00	2	1.26
Speed of daily tasks (DAS-M13, n = 21)	2.05	2	1.02
Communication and social contacts (DAS-M14, n = 21)	2.05	2	0.97
Mastering social conflicts (DAS-M15)	0.48	0	0.93
Mastering emergency situations (DAS-M16, n = 21)	0.90	1	1.04
Functioning in household and family (DAS-M21, n = 19)	1.79	2	1.23
Sexual role functioning (DAS-M25, n = 18)	2.17	2.5	1.42
Work functioning (DAS-M26, n = 19)	2.47	3	0.96
Motivation to work (DAS-M27, n = 20)	1.70	1.5	1.26
Functioning as citizen and consumer (DAS-M28, n = 21)	2.00	2	1.38
Overall social functioning (DAS-M3G, n = 21)	3.05	3	0.86
*Cognitive functioning (WAIS*, *n = 25)*			
Verbal IQ score	99.2	101	10.5
Nonverbal IQ score	100.8	100	11.0
Total IQ score	100.0	98	9.6
*Self concept (FSKN*, *n = 26)*			
Total score	315.2	307.5	59.6
*Coping with Stress (SVF)*			
Negative coping ^c^	10.4	10.5	4.3
Total score	224.7	212	57.7

^a^ Factor scores according to Kay et al. [55]

^b^ Higher scores imply higher social competence, z-transformed sum scores for nonverbal social skills not listed

^c^ Mean score of SVF subscales 13 to 18

## Results

### Synchrony versus Pseudosynchrony

The comparison of genuine synchrony to pseudosynchrony indicated that the phenomenon of nonverbal synchrony was present at a highly significant level and with a medium to high effect-size (*t* = 4.91, *p* < .001, Cohen's *d* = 0.7, *n* = 27). The amount of nonverbal coordination thus clearly exceeded the levels one would expect if the coordination was only due to chance.

### Correlations Between Nonverbal Synchrony and Symptom Factors

Head synchrony was weakly correlated with amount of movement (*r* = .25, n.s.), indicating that, importantly, neither measure was redundant. Head synchrony was similar in male and female patients (*t* = 0.27, n.s., Cohen's *d* = 0.13, *n* = 27), and it was correlated weakly with age (*r* = .20, n.s.). Correlations between head synchrony and PANSS symptom factors are reported in Table 2. Nonverbal synchrony showed significant negative associations with negative symptoms, the cognitive factor (including symptoms of disorganization), the depression factor, and total PANSS scores.

**Table 2:** Correlations of head synchrony and PANSS symptom factors and PANSS total score (n = 27).

Symptom factors	Head synchrony^a^	Head movement^c^	Head synchrony residuals^d^	Patient imitates movements	Patient’s movements are imitated
Negative factor	-.42 *	-.51 **	-.33 ^†^	-.46 *	-.26
Positive factor	-.29	.11	-.32	-.13	-.47 *
Cognitive factor	-.46 * ^b^	-.07	-.45 *	-.35 ^†^	-.38 *
Excitement factor	-.21	-.32 ^†^	-.14	-.13	-.25
Depression factor	-.43 *	-.31	-.37 ^†^	-.48 *	-.36 ^†^ ^a^
PANSS total	-.53 ** ^b^	-.37 ^†^	-.47 *	-.49 **	-.46 *

^a^ Correlations with head synchrony (first column) were the main calculations for this study. Other calculations were exploratory.

^b^Relationship remained significant with α = 0.05 after applying the Benjamini and Yekutieli false discovery rate (FDR) control procedure [70] for *k* = 6 pairwise tests.

^c^Head movement = proportion of time moving (patient)

^d^ Head synchrony corrected for the amount of head movement

† *P <* .1.

* *P <* .05.

** *P <* .01.

The middle column of Table 2 provides correlations between patients' head movements and symptom scores [35]. Residual correlations between head synchrony and symptoms, corrected for the amount of patients' head movements, are reported in the third column of Table 2. When correcting for head movement, residual head synchrony was still consistently related to symptom factors. Correlations between residual head synchrony and symptom factors ranged from -.32 to -.47, except for the excitement factor. Both head synchrony and residual head synchrony, but not patient's head movement, were significantly related to the cognitive factor. Negative symptoms were negatively correlated with imitation, whereas positive symptoms were positively correlated with being imitated. Relationships between all movement variables and symptoms were substantial.

### Correlations Between Nonverbal Synchrony and Symptoms

To further explore relationships between head synchrony and symptoms, we calculated correlations of the two movement variables with the individual symptoms included in the five PANSS factors (Table 3). Given the high number of correlations based on a relatively small sample size, the calculations provided in Table 2 should be considered exploratory.

**Table 3:** Correlations of nonverbal synchrony and PANSS symptoms (n = 27).

Symptoms	Head synchrony^a^	Head movement^c^	Head synchrony residuals^d^	Patient imitates movements	Patient’s movements are imitated
Negative symptoms					
Blunted affect (N1)	-.53 ** ^d^	-.41 *	-.47 *	-.52 **	-.22
Emotional withdrawal (N2)	-.32	-.60 ***	-.22	-.38 ^†^	- .20
Poor Rapport (N3)	-.32	-.31	-.26	-.32	-.25
Passive/apathetic social withdrawal (N4)	-.35 ^†^	-.38 ^†^	-.28	-.31	-.44 *
Lack of spontaneity/ conversation flow (N6)	-.08	-.26	-.02	-.27	.15
Active social avoidance (G16)	-.22	-.32 ^†^	-.14	-.26	-.20
Positive symptoms					
Delusions (P1)	-.21	.18	-.26	-.03	-.31
Hallucinatory behavior (P3)	.25	.06	.24	.18	.14
Grandiosity (P5)	-.13	.29	-.22	.04	-.39*
Suspiciousness/ persecution (P6)	-.47 *	-.37 ^†^	-.40 *	-.41*	-.46 *
Unusual thought content (G9)	-.18	.35 ^†^	-.28	-.02	-.31
Cognitive symptoms					
Conceptual disorganization (P2)	-.49 * ^b^	-.06	-.47 *	-.38 ^†^	-.34 ^†^
Difficulty in abstract thinking (N5)	-.12	.08	-.13	.02	-.25
Disorientation (G10)	.00	.00	.00	.00	.00
Mannerisms and posturing (G5)	-.26	.10	-.28	-.20	-.24
Poor attention (G11)	-.27	-.30	-.21	-.32	-.09
Excitement symptoms					
Excitement (P4)	.26	-.12	.29	.18	.22
Hostility (P7)	-.27	-.29	-.20	-.31	-.25
Tension (G4)	-.34 ^†^	-.29	-.28	-.25	-.25
Poor impulse control (G14)	-.23	-.17	-.19	.02	-.39 *
Depression symptoms					
Somatic concern (G1)	-.11	-.04	-.10	-.22	-.06
Anxiety (G2)	-.13	.03	-.13	-.10	-.15
Guilt feeling (G3)	-.37 ^†^	-.23	-.32	-.33 ^†^	- .34 ^†^
Depression (G6)	-.26	-.36 ^†^	-.18	-.34 ^†^	-.20
Preoccupation (G15)	-.37 ^†^	-.23	-.32	-.36 ^†^	-.26
Other symptoms					
Stereotyped thinking (N7)	-.30	-.04	-.29	-.23	-.28
Motor retardation (G7)	-.25	-.30	-.18	-.35	.02
Lack of judgment/insight (G12)	-.43 *	-.16	-.39 *	-.27	-.46 *
Disturbance of volition (G13)	-.14	-.27	-.08	-.23	-.08

^a^ Correlations with head synchrony (first column) were the main calculations for this study. Other calculations were exploratory.

^b^Relationship remained significant with α = 0.05 after applying the Benjamini and Yekutieli false discovery rate (FDR) control procedure [70] for *k* = 29 pairwise tests.

^c^Head movement = proportion of time moving (patient)

^d^ Head synchrony corrected for the amount of head movement

† *P <* .1.

* *P <* .05.

** *P <* .01

*** *P <* .001.

Consistent with the correlations between head synchrony and the symptom factors presented in Table 2, moderate to strong associations between head synchrony and individual PANSS symptoms were observed. Relationships between symptom factors and synchrony remained significant in a number of instances, even when correcting for amounts of head movement (e.g., for symptom N1, "blunted affect" and symptom P6, "suspiciousness/persecution"). In the latter case, results suggested that suspiciousness was independently related to both reduced head movement and reduced synchrony of the movements. Other symptoms were specifically related to movement and less strongly associated with head synchrony (e.g., symptom N2 "emotional withdrawal").

Results suggested that head synchrony is selectively related to particular symptoms. Some relationships between head synchrony and symptoms differed considerably from the relationships between amounts of movement and symptoms. Lower synchrony was specifically related to conceptual disorganization (symptom P2 of the PANSS), and movement and head synchrony were not associated with difficulty in abstract thinking. Lack of judgment and insight (symptom G12 of the PANSS) were related to lower levels of head synchrony. Once again, differential relationships between symptom factors and the two components of synchrony were observed: "blunted affect," symptom N1, was negatively associated with imitation, whereas "grandiosity" was negatively related to being imitated. Patients with higher scores in negative symptoms did not imitate their interaction partners, while patients with high scores in positive symptoms were not imitated by their interaction partners.

### Correlations Between Nonverbal Synchrony and Social Competence and Functioning

Table 4 summarizes the correlations between movement and coordination variables and social competence in the RPT. Social competence was rated by independent raters, who were unaware of any clinical data and results. Head synchrony was significantly related to the global score for the RPT, nonverbal social skills, and gaze/eye contact. Correcting for head movement, associations remained significant or tended to be significant. Again, there were indications of specific associations between head synchrony and social skills. Head synchrony was specifically associated with gaze and eye contact, and to some extent, meshing/latency but not to speech duration. The global score of social competence was considerably associated with patients' being less imitated by their interaction partners.

**Table 4:** Correlations: nonverbal synchrony and rated social competence in the role play test (n = 27).

Social skills scores	Head synchrony^a^	Head movement^c^	Head synchrony residuals^d^	Patient imitates movements	Patient’s movements are imitated
Global score	.47 * ^b^	.30	.36 ^†^	-.32	-.50 **
Nonverbal social skills	.40 *	.23	.32 ^†^	-.36 ^†^	-.25
Gaze / eye contact	.41*	.04	.40 *	-.33	-.28
Affect	.27	.35 ^†^	.20	-.31	-.30
Speech duration	.13	.38 ^†^	.04	-.13	-.04
Meshing/latency	.35 ^†^	-.04	.36 ^†^	-.27	-.12

^a^ Correlations with head synchrony (first column) were the main calculations for this study. Other calculations were exploratory.

^b^Relationship remained significant with α = 0.05 after applying the Benjamini and Yekutieli false discovery rate (FDR) control procedure [70] for *k* = 6 pairwise tests.

^c^Head movement = proportion of time moving (patient)

^d^ Head synchrony corrected for the amount of head movement

† *P <* .1.

* *P <* .05.

** *P <* .01.

Detailed reports on social impairment, based on observations of family or professional informants using the DAS-M scale [60], were available for 21 of the 27 patients (Table 5).

**Table 5:** Correlations: Nonverbal synchrony and social impairment according to the Disability Assessment Schedule, Mannheim form (DAS-M).

Domain of social functioning	Head synchrony^a^	Head movement^c^	Patient imitates movements	Patient’s movements are imitated
Self-care and personal presentation (DAS-M11, *n* = 21)	-.35	-.12	-.08	-.40 ^†^
Leisure activities (DAS-M12, *n* = 21)	-.28	-.15	-.23	-.42 ^†^
Speed of daily tasks (DAS-M13, *n* = 21)	-.21	-.11	-.21	-.20
Communication and social contacts (DAS-M14, *n* = 21)	-.34	-.22	-.31	-.57 **
Mastering social conflicts (DAS-M15)	.02	-.09	.30	-.25
Mastering emergency situations (DAS-M16, *n* = 21)	-.36	-.04	-.28	-.43 ^†^
Functioning in household and family (DAS-M21, *n* = 19)	-.37	-.07	-.09	-.63 **
Sexual role functioning (DAS-M25, *n* = 18)	-.39	.23	-.16	-.74 ***
Work functioning (DAS-M26, *n* = 19)	-.19	-.33	-.03	-.48 *
Motivation to work (DAS-M27, *n* = 20)	-.48 * ^b^	-.30	-.29	-.60 *
Functioning as citizen and consumer (DAS-M28, *n* = 21)	-.31	-.10	-.07	-.34
Overall social functioning (DAS-M3G, *n* = 21)	-.36	-.30	-.09	-.53 *

^a^ Correlations with head synchrony (first column) were the main calculations for this study. Other calculations were exploratory.

^b^Relationship remained significant with α = 0.05 after applying the Benjamini and Yekutieli false discovery rate (FDR) control procedure [70] for *k* = 12 pairwise tests.

^c^Head movement = proportion of time moving (patient)

† *P <* .1.

* *P <* .05.

** *P <* .01

*** *P <* .001.

There was no indication of any association between raw amounts of head movement and social impairment in any of the DAS-M scales (see second column); therefore, no residual relationships were calculated. Head synchrony correlated negatively with impaired general social adaptation (*r* = -.36, *p* = .11, *n* = 21). The subscales of the DAS-M showed similar associations between head synchrony and social impairment. Impaired "motivation to work," subscale 2–7 of the DAS-M, showed the strongest negative association with head synchrony (*r* = -.48, *p* = .03, *n* = 21). In addition, there was no indication of any association between amount of movement and social impairment in any of the DAS-M scales. Social impairment, as measured by the DAS-M, was strongly negatively associated with being imitated (i.e., the tendency of interaction partners to imitate patients’ movements).

### Correlations Between Nonverbal Synchrony and Cognitive Variables

There was no association between head synchrony and full scale IQ of the WAIS intelligence test (*r* = .09, *p* = .68, *n* = 25). Neither amount of movement nor head synchrony was associated with verbal or performance IQ in the WAIS. However, head synchrony was associated with performance in the WAIS digit span test (*r* = .53, *p* = .006, *n* = 25). Digit span assesses aspects of attention and short-term verbal memory. Head synchrony was associated with the total score for the FSKN, assessing beliefs regarding competence in diverse domains of daily functioning (*r* = .42, *p* = .03, *n* = 26). Head synchrony was not correlated with the SVF total score, but correlated negatively with a self-assessment of "negative coping" in the SVF questionnaire (*r* = -.37, *p* = .05, *n* = 27). Negative coping refers to an evasive and ruminative style of coping with challenging situations [73].

## Discussion

We examined the importance of head synchrony emerging during social interaction of patients with schizophrenia and healthy interactants. Specifically, using video recordings of role-play tests, the relationships between head synchrony and patients' symptoms, as well as other signs of the disorder, were analyzed. Results showed that lower head synchrony between patients and their interactants was indicative of patients’ symptoms (negative symptoms in addition to conceptual disorganization and lack of insight, as assessed using the PANSS), low social competence, impaired social functioning, and patients' low self-evaluation of competence. These relationships remained significant even when correcting for the relative amounts of patients’ head movement, suggesting that head synchrony provides additional and specific information regarding patients’ functioning over and above psychomotor retardation. Generally, head synchrony was more strongly associated with symptoms and other variables than patients' extent of head movement alone. Exploratory calculations suggested that the direction of nonverbal synchrony (patient imitating vs. being imitated) was differentially related to several symptoms and specifically related to social functioning. Reduced synchrony, based on a lack of patients’ imitation of their interactants, was related to negative symptoms, whereas a lack of imitation of patients by their interactants was related to the positive symptom factor. The latter reduction in imitation was most strongly associated with impaired social functioning, particularly in domains where social interaction is crucial (communication, household functioning, and sexual role functioning).

Further calculations suggested that head synchrony was not generally associated with indicators of cognitive deficit. However, an association was observed between reduced synchrony and verbal short-term memory, which is congruous with the association between reduced head synchrony and conceptual disorganization. Overall, the exploratory calculations gave a consistent picture of associations between head synchrony and symptoms, cognition, and social functioning was observed, supporting the notion that head synchrony may in fact indicate and differentiate core deficits in social functioning.

### Particularities of synchrony in patients with schizophrenia

Nonverbal synchrony is a facet of social interaction with a complex set of determinants [41]. Results in this sample of schizophrenia patients suggested that reduced nonverbal synchrony was related to two larger domains of functioning and symptoms: Nonverbal synchrony may be compromised by affective and motivational problems, which are often associated with overall impairments in motor activity [74], and more specifically with changes in e.g. nonverbal expressivity [75,76], and the presence of negative symptoms [35]. Additionally, as suggested by the analysis of residual synchrony, synchrony may be reduced by cognitive problems, with conceptual disorganization and deficits in short-term verbal memory as prominent markers of impairment.

The most notable finding of this present study was the specific association between patient's imitation of the interactant and the patient being imitated by the interactant: Imitating and being imitated reveal separate aspects of nonverbal interaction, namely a differentiation into the two categories of imitating the healthy participant and being imitated by the healthy participant. In this sample, patients' negative symptoms were associated with less imitation occurring on patients' side. Such a lower level of imitative behavior could be explained either by deficits in their decoding of nonverbal information–patients are unable to "register" nonverbal cues, or, by deficits in the expression of nonverbal information–patients are unable to "react to" nonverbal cues of others. Experimental research mainly confirms the latter: Patients with negative symptoms perform worse regarding their expressive behavior [18]. Moreover, they perform worse in empathic accuracy, implying that the tracking of emotional states of others is impaired [77]. For positive symptoms, patients' reduced social functioning was associated with less imitation occurring on the healthy participants' side: This lower level of being imitated could be explained by deficits in encoding nonverbal information–patients are unable to "send" adequate nonverbal cues. The inadequacy might be due to inappropriate or deviant amounts of nonverbal behavior, resulting from psychotic states of mind. Our own previous study hints at the latter: Patients suffering from positive symptoms generally displayed higher amounts of head movement [35].

In other words, depending on their symptomatology, patients either weren't able to *react* to nonverbal behavior themselves (they didn't imitate), or they *displayed* abnormal nonverbal behavior, that their interaction partners didn't react to (they weren't imitated). Such a distinction has not yet received a lot of attention in the literature, because the majority of studies have focused on the patient as the object of investigation and not on the dyad or group as an entity. The differential associations described above point to an important facet inherent in social nonverbal behavior: The behaviors displayed should better not be analyzed in isolation, because important facets of nonverbal behavior only emerge in the course of multiple individuals' *interaction*. A similar conclusion has been reached in an experimental study comparing empathic accuracy in schizophrenia patients, which provided evidence for reduced empathy in patients [77]. The exact mechanism of the differential reduction described in this study should be clarified in future research; the reduction of imitation by interactants has already been demonstrated in experimental research [21].

Various sources have suggested that the lack of synchrony in schizophrenia patients may be a crucial factor influencing their performance in social behavior [4,12,13,21,22]. The associations between lower synchrony and lower social functioning reported here, are in line with recent work on the experimental variation of intentional social coordination [21,74] in an experimental setting [22], and with unintentional social coordination in a triadic setting involving one patient and two healthy interactants [36,53]. Our distinction of *imitating* and *being imitated* provides further data to the list of possible obstacles arising in nonverbal communication of schizophrenic patients [78,79].

### Possible Applications

To our knowledge, the present study is one of the few studies using video-based objective quantification of synchrony in dyadic social interactions of patients with schizophrenia. The central role of this characteristic for social functioning makes it a prime target for future interventions: It has already been shown that the deliberate aspect of this property may be susceptible to e.g. subliminal priming [21] and later lead to a better relationship quality with an interaction partner. The differential pathways leading to the patterns described above should be explored further, as they have implications for therapeutic interventions aimed at increasing social functioning in patients with schizophrenia. Our results corroborate the behavioral treatment rationale increasing both decoding/perception and encoding/expression in patients with negative symptoms, which is done in e.g. IPT treatments [80]. Furthermore, these results are congruent with cognitive treatment approaches for cognitive symptoms [81]. In these approaches, cognitive processes underlying distorted processes of perception and behavior are targeted.

### Limitations

Given the small sample size, the relationships between head synchrony and the five symptom domains of the PANSS may be understood as the main result of the present study; additional relationships reported should be considered exploratory. The short, more restricted, and experimental nature of the role-play interactions used in the present study is different from non-scripted social interaction. It is possible that the nature of the role-play tests allowed for less interactional adaptation [3]. Given the unavailability of a reference group of healthy interactants, we applied a bootstrapping approach [70] to compare the general levels of synchrony to the levels of synchrony one would expect to observe by chance. This comparison revealed that nonverbal synchrony was clearly distinguishable from random coincidence. The attained effect-size of *d* = 0.7 lies within the range previously found in other interaction settings: Effect-sizes for psychotherapy dyads [41] were lower (0.5 to 0.6) whereas discussions between healthy students resulted in higher scores (0.7 to 1.2) [48]. The lower effect-size found in psychotherapy likely reflects the fact that psychotherapeutic interactions are much less structured in comparison to the role-plays employed here and to the socio-political discussions between healthy students.

This implies that the use of staged role-play interactions may be both a limitation and an asset of the present study. It limits generalizability to less structured social interactions such as unscripted social encounters or the less predictable flow of verbal exchange in psychotherapy. However, the high degree of standardization, which make such role play interactions powerful, reliable, and valid tests [57], may also have been helpful for identifying the observed relationships. The present study is based on data originally collected in a study on the rehabilitation of patients with schizophrenia [73,82–84]. Accordingly, the patient group was restricted to a relatively stabilized group of patients with schizophrenia. A wide variety of measures from the original study, including measures of social functioning, were reported in order to provide a comprehensive description of the patients. Furthermore, this facilitated the identification of possible associations between nonverbal synchrony and social and cognitive functioning.

### Future Research

In further research, analysis of nonverbal synchrony and imitation should be complemented by measures of social cognition to explore relationships between nonverbal synchrony and social information processing. On a neurophysiological level, the association of nonverbal synchrony with alterations in the brain regions and circuits involved in the human mirroring system [12,85,86] provides promising targets. Intensified research on nonverbal synchrony, as researching movement patterns in general [14,16], may have additional appeal for translational research on mental disorders, due to the pervasive importance of movement and socio-communicative movement patterns in humans and animals. Research on nonverbal synchrony may help to integrate currently distant concepts, such as Bleulers’ "autism" in schizophrenia [29] or Rümke’s "praecox feeling" [27], with research on social cognition or the mirroring system’s contribution to schizophrenic disorders. In this way, quantitative research on nonverbal behavior in schizophrenia may help building bridges between clinical knowledge and basic research.

## References

[1] Segerstråle U, Molnár P. Nonverbal communication: Where nature meets culture Segerstråle U, Molnár P, editors. Mahwah, NJ: Lawrence Erlbaum; 1997.

[2] Riggio RE, Feldman RS. Applications of nonverbal communication Riggio RE, Feldman RS, editors. Mahwah, NJ: Lawrence Erlbaum Associates Publishers; 2005.

[3] Burgoon JK, Stern LA, Dillman L. Interpersonal adaptation: Dyadic interaction patterns Cambridge: Cambridge University Press; 1995.

[4] Condon WS, Ogston WD. Sound film analysis of normal and pathological behavior patterns. J Nerv Ment Dis 1966;143(4):338–457. 5958766 10.1097/00005053-196610000-00005

[5] Leclère C, Viaux S, Avril M, Achard C, Chetouani M, Missonnier S, et al Why synchrony matters during mother-child interactions: A systematic review. PLoS ONE 2014;9(12):e113571 10.1371/journal.pone.0113571 25469637 PMC4254467

[6] Chartrand TL, Lakin JL. The antecedents and consequences of human behavioral mimicry. Annu Rev Psychol 2013;64:285–308. 10.1146/annurev-psych-113011-143754 23020640

[7] Latif N, Barbosa AV, Vatiokiotis-Bateson E, Castelhano MS, Munhall KG. Movement coordination during conversation. PLoS ONE 2014;9(8):e105036 10.1371/journal.pone.0105036 25119189 PMC4132081

[8] Gallese V, Fadiga L, Fogassi L, Rizzolatti G. Action recognition in the premotor cortex. Brain 1996;119:593–609. 8800951 10.1093/brain/119.2.593

[9] Rizzolatti G, Fadiga L, Gallese V, Fogassi L. Premotor cortex and the recognition of motor actions. Cog Brain Res 1996;3(2):131–41. 10.1016/0926-6410(95)00038-0 8713554

[10] Iacoboni M. Imitation, empathy, and mirror neurons. Annu Rev Psychol 2009;60:653–70. 10.1146/annurev.psych.60.110707.163604 18793090

[11] Voelkl B, Huber L. Imitation as faithful copying of a novel technique in marmoset monkeys. PLoS ONE 2007;2(7):e611 17622356 10.1371/journal.pone.0000611 PMC1905941

[12] Gallese V, Sinigaglia C. What is so special about embodied simulation? Trends Cogn Sci 2011;15(11):512–9. 10.1016/j.tics.2011.09.003 21983148

[13] Mehta UM, Thirthalli J, Basavaraju R, Gangadhar BN, Pascual-Leone A. Reduced mirror neuron activity in schizophrenia and its association with theory of mind deficits: Evidence from a transcranial magnetic stimulation study. Schizophr Bull 2014;40(5):1083–94. 10.1093/schbul/sbt155 24214933 PMC4133670

[14] Young JW, Minassian A, Paulus MP, Geyer MA, Perry W. A reverse-translational approach to bipolar disorder: Rodent and human studies in the behavioral pattern monitor. Neurosci Biobehav Rev 2007;31(6):882–96. 17706782 10.1016/j.neubiorev.2007.05.009 PMC2025688

[15] Walther S, Horn H, Koschorke P, Müller TJ, Strik W. Increased motor activity in cycloid psychosis compared to schizophrenia. World J Biol Psychiat 2009;10(4 Pt 3):746–51. 10.1080/15622970701882425 18609426

[16] Walther S, Ramseyer F, Horn H, Strik W, Tschacher W. Less structured movement patterns predict severity of positive syndrome, excitement, and disorganization. Schizophr Bull 2014;40(3):585–91. 10.1093/schbul/sbt038 23502433 PMC3984503

[17] Del-Monte J, Raffard S, Salesse RN, Marin L, Schmidt RC, Varlet M, et al Nonverbal expressive behaviour in schizophrenia and social phobia. Psychiat Res 2013;210(1):29–35. 10.1016/j.psychres.2013.05.034 23845416

[18] Brüne M, Sonntag C, Abdel-Hamid M, Lehmkämper C, Juckel G, Troisi A. Nonverbal behavior during standardized interviews in patients with schizophrenia spectrum disorders. J Nerv Ment Dis 2008;196(4):282–8. 10.1097/NMD.0b013e31816a4922 18414122

[19] Andreasen NC, Grove WM. Thought, language, and communication in schizophrenia: Diagnosis and prognosis. Schizophr Bull 1986;12(3):348 3764356 10.1093/schbul/12.3.348

[20] Meyer EC, Carrión RE, Cornblatt BA, Addington J, Cadenhead KS, Cannon TD, et al The relationship of neurocognition and negative symptoms to social and role functioning over time in individuals at clinical high risk in the first phase of the north american prodrome longitudinal study. Schizophr Bull 2014;40(6):1452–61. 10.1093/schbul/sbt235 24550526 PMC4193704

[21] Raffard S, Salesse RN, Marin L, Del-Monte J, Schmidt RC, Varlet M, et al Social priming enhances interpersonal synchronization and feeling of connectedness towards schizophrenia patients. Sci Rep 2015;5:8156 10.1038/srep08156 25640605 PMC4313116

[22] Varlet M, Marin L, Raffard S, Schmidt RC, Capdevielle D, Boulenger JP, et al Impairments of social motor coordination in schizophrenia. PLoS ONE 2012;7(1):e29772 10.1371/journal.pone.0029772 22272247 PMC3260163

[23] Giacco D, McCabe R, Kallert T, Hansson L, Fiorillo A, Priebe S. Friends and symptom dimensions in patients with psychosis: A pooled analysis. PLoS ONE 2012;7(11):e50119 10.1371/journal.pone.0050119 23185552 PMC3503760

[24] Schmidt SJ, Mueller DR, Roder V. Social cognition as a mediator variable between neurocognition and functional outcome in schizophrenia: Empirical review and new results by structural equation modeling. Schizophr Bull 2011;37 Suppl 2:S41–54. 10.1093/schbul/sbr079 21860046 PMC3160114

[25] Green MF, Leitman DI. Social cognition in schizophrenia. Schizophr Bull 2008;34(4):670–2. 10.1093/schbul/sbn045 18495642 PMC2632454

[26] Hill D. Non-verbal behaviour in mental illness. Brit J Psychiat 1974; 124:221–30. 4600313 10.1192/bjp.124.3.221

[27] Rümke HC. Das Kernsyndrom der Schizophrenie und das ‘Praecox-Gefühl’. Zentralblatt Gesamte Neurologie und Psychiatrie 1941;102:168–9.

[28] Parnas J. A disappearing heritage: The clinical core of schizophrenia. Schizophr Bull 2011;37(6):1121–30. 10.1093/schbul/sbr081 21771902 PMC3196960

[29] Bleuler E. Dementia praecox oder die Gruppe der Schizophrenien. Leipzig: Deuticke; 1911. 10.1192/bjp.149.5.661 3545358

[30] Gaebel W, Wölwer W. Facial expressivity in the course of schizophrenia and depression. Eur Arch Psychiat Clin Neurosci 2004;254(5):335–42. 10.1007/s00406-004-0510-5 15365710

[31] Kohler CG, Martin EA, Stolar N, Barrett FS, Verma R, Brensinger C, et al Static posed and evoked facial expressions of emotions in schizophrenia. Schizophr Res 2008;105(1):49–60. 18789845 10.1016/j.schres.2008.05.010 PMC5048468

[32] Mattes RM, Schneider F, Heimann H, Birbaumer N. Reduced emotional response of schizophrenic patients in remission during social interaction. Schizophr Res 1995;17(3):249–55. 8664204 10.1016/0920-9964(95)00014-3

[33] Trémeau F, Malaspina D, Duval F, Corrêa H, Hager-Budny M, Coin-Bariou L, et al Facial expressiveness in patients with schizophrenia compared to depressed patients and nonpatient comparison subjects. Am J Psychiat 2005;162(1):92–101. 15625206 10.1176/appi.ajp.162.1.92

[34] Brüne M, Sonntag C, Abdel-Hamid M, Lehmkämper C, Juckel G, Troisi A. Nonverbal behavior during standardized interviews in patients with schizophrenia spectrum disorders. J Nerv Ment Dis 2008;196(4):282–8. 10.1097/NMD.0b013e31816a4922 18414122

[35] Kupper Z, Ramseyer F, Hoffmann H, Kalbermatten S, Tschacher W. Video-based quantification of body movement during social interaction indicates the severity of negative symptoms in patients with schizophrenia. Schizophr Res 2010;121(1–3):90–100. 10.1016/j.schres.2010.03.032 20434313

[36] Lavelle M, Healey PG, McCabe R. Is nonverbal communication disrupted in interactions involving patients with schizophrenia? Schizophr Bull 2013;39(5):1150–8. 10.1093/schbul/sbs091 22941744 PMC3756773

[37] Lavelle M, Healey PG, McCabe R. Nonverbal behavior during face-to-face social interaction in schizophrenia: A review. J Nerv Ment Dis 2014;202(1):47–54. 10.1097/NMD.0000000000000031 24375212

[38] Lavelle M, Healey PG, McCabe R. Participation during first social encounters in schizophrenia. PLoS ONE 2014;9(1):e77506 10.1371/journal.pone.0077506 24465363 PMC3896339

[39] Ramseyer F, Tschacher W. Synchrony: A core concept for a constructivist approach to psychotherapy. Construct Hum Sci 2006;11(1–2):150–71.

[40] Ramseyer F, Tschacher W. Synchrony in dyadic psychotherapy sessions In: Vrobel S, Rössler OE, Marks-Tarlow T, editors. Simultaneity: Temporal Structures and Observer Perspectives. Singapore: World Scientific; 2008 p. 329–47.

[41] Ramseyer F, Tschacher W. Nonverbal synchrony in psychotherapy: Coordinated body-movement reflects relationship quality and outcome. J Consult Clin Psychol 2011;79(3):284–95. 10.1037/a0023419 21639608

[42] Ramseyer F, MEA [computer program]; 2008 University of Bern, Switzerland http://www.psync.ch.

[43] Altmann U. Investigation of movement synchrony using windowed cross-lagged regression. In: Esposito A, Vinciarelli A, Vicsi K, Pelachaud C, Nijholt A, editors. Analysis of Verbal and Nonverbal Communication and Enactment. The Processing Issues.; 2011. p. 335–45.

[44] Grammer K, Honda R, Schmitt A, Jütte A. Fuzziness of nonverbal courtship communication unblurred by motion energy detection. J Pers Soc Psychol 1999;77(3):487–508. 10510505 10.1037//0022-3514.77.3.487

[45] Delaherche E, Chetouani M, Mahdaoui A, Saint-Georges C, Viaux S, Cohen D. Interpersonal synchrony: A survey of evaluation methods across disciplines. IEEE Transactions on Affective Computing 2012;3(3):349–65.

[46] Paxton A, Dale R. Frame-differencing methods for measuring bodily synchrony in conversation. Behav Res Meth 2013;45(2):329–43. 10.3758/s13428-012-0249-2 23055158

[47] Nelson A, Grahe J, Ramseyer F, Serier K. Psychological data from an exploration of the rapport/synchrony interplay using motion energy analysis. J Open Psychol Data 2014;2(1):e5.

[48] Tschacher W, Rees GM, Ramseyer F. Nonverbal synchrony and affect in dyadic interactions. Front Psychol 2014;5:1323 10.3389/fpsyg.2014.01323 25505435 PMC4241744

[49] Scheflen AE. Communicational structure: Analysis of a psychotherapy transaction Bloomington: Indiana University Press; 1973.

[50] Hardin SB. Comparative analysis of nonverbal interpersonal communication of schizophrenics and normals. Res Nurs Health 1980;3(2):57–68. 6901546 10.1002/nur.4770030204

[51] Ellgring H. Nonverbal expression of psychological states in psychiatric patients. Eur Arch Psychiat Neurol Sci 1986;236(1):31–4. 10.1007/BF00641055 3743583

[52] Pounds KG. Client-nurse interaction with individuals with schizophrenia: A descriptive pilot study. Iss Ment Health Nurs 2010;31(12):770–4. 10.3109/01612840.2010.518337 21142597

[53] Lavelle M. Nonverbal communication in schizophrenia: A 3-D Analysis of patients’ social interactions [Doctoral dissertation]. London, UK: Queen Mary, University of London; 2011.

[54] Del-Monte J, Capdevielle D, Varlet M, Marin L, Schmidt RC, Salesse RN, et al Social motor coordination in unaffected relatives of schizophrenia patients: A potential intermediate phenotype. Front Behav Neurosci 2013;7:137 10.3389/fnbeh.2013.00137 24106467 PMC3788352

[55] Kay SR, Flszbein A, Opfer LA. The positive and negative syndrome scale (PANSS) for schizophrenia. Schizophr Bull 1987;13(2):261–76. 3616518 10.1093/schbul/13.2.261

[56] Lindenmayer J-P, Grochowski S, Hyman RB. Five factor model of schizophrenia: Replication across samples. Schizophr Res 1995;14(3):229–34. 7766534 10.1016/0920-9964(94)00041-6

[57] Bellack AS, Brown CH, Thomas-Lohrman S. Psychometric characteristics of role-play assessments of social skill in schizophrenia. Behav Ther 2006;37(4):339–52. 17071212 10.1016/j.beth.2006.01.005

[58] Kupper Z, Käser I, Kunz B, Hoffmann H. Soziale Kompetenz bei schizophrenen Patienten. Deutsche Adaptation des interaktiven Rollenspieltests (IRST). Z Klin Psychol Psychiatr Psychother 2004;52(1):59–77.

[59] Bellack AS, Morrison RL, Mueser KT, Wade JH, Sayers SL. Role play for assessing the social competence of psychiatric patients. Psychol Assess 1990;2(3):248.

[60] Jung E, Krumm B, Biel H, Maurer K, Bauer-Schubart C. Disability assessment schedule (DAS-M): Mannheimer Skala zur Einschätzung sozialer Behinderung. Weinheim: Beltz; 1989.

[61] Wechsler D. Die Messung der Intelligenz Erwachsener: Textband zum Hamburg-Wechsler-Intelligenztest für Erwachsene (HAWIE). Bern: Huber; 1964.

[62] Deusinger IM. Die Frankfurter Selbstkonzeptskalen:(FSKN). Göttingen: Hogrefe; 1986.

[63] Janke W, Erdmann G, Boucsein W. Stressverarbeitungsfragebogen (SVF). Göttingen: Hogrefe; 1985.

[64] Boker SM, Xu M, Rotondo JL, King K. Windowed cross-correlation and peak picking for the analysis of variability in the association between behavioral time series. Psychol Methods 2002;7(3):338–55. 12243305 10.1037/1082-989x.7.3.338

[65] Derrick TR, Thomas JM. Time series analysis: The cross-correlation function In: Stergiou N, editors. Innovative analyses of human movement. Champaign, IL: Human Kinetics; 2004 p. 189–205.

[66] Grammer K, Kruck KB, Magnusson MS. The courtship dance: Patterns of nonverbal synchronization in opposite-sex encounters. J Nonverbal Behav 1998;22(1):3–29.

[67] McGarva A, Warner R. Attraction and social coordination: Mutual entrainment of vocal activity rhythms. Journal of Psycholinguistic Research 2003;32(3):335–54. 12845943 10.1023/a:1023547703110

[68] Bernieri FJ, Rosenthal R. Interpersonal coordination: Behavior matching and interactional synchrony In: Feldman RS, Rime B, editors. Fundamentals of nonverbal behavior. Studies in emotion & social interaction New York: Cambridge University Press; 1991 p. 401–32.

[69] Bernieri FJ, Reznick S, Rosenthal R. Synchrony, pseudosynchrony, and dissynchrony: Measuring the entrainment process in mother-infant interactions. J Pers Soc Psychol 1988;54(2):243–53.

[70] Ramseyer F, Tschacher W. Nonverbal synchrony or random coincidence? How to tell the difference In: Esposito A, Campbell N, Vogel C, Hussain A, Nijholt A, editors. Development of Multimodal Interfaces: Active Listening and Synchrony. Berlin: Springer; 2010 p. 182–96.

[71] Benjamini Y, Yekutieli D. The control of the false discovery rate in multiple testing under dependency. Ann Stat 2001;29:1165–88.

[72] Narum SR. Beyond Bonferroni: less conservative analyses for conservation genetics, Conserv Genet 2006;7:783–7.

[73] Hoffmann H, Kupper Z, Kunz B. Hopelessness and its impact on rehabilitation outcome in schizophrenia—an exploratory study. Schizophr Res 2000;43(2):147–58. 10858633 10.1016/s0920-9964(99)00148-6

[74] Morrens M, Docx L, Walther S. Beyond boundaries: In search of an integrative view on motor symptoms in schizophrenia. Front Psychiat 2014;5:145. 10.3389/fpsyt.2014.00145 PMC4196470 25352812

[75] Annen S, Roser P, Brüne M. Nonverbal behavior during clinical interviews: Similarities and dissimilarities among schizophrenia, mania, and depression. J Nerv Ment Dis 2012;200(1):26–32. 10.1097/NMD.0b013e31823e653b 22210359

[76] Walther S, Stegmayer K, Sulzbacher J, Vanbellingen T, Müri R, Strik W, et al Nonverbal social communication and gesture control in schizophrenia. Schizophr Bull 2015;41(2):338–45. 10.1093/schbul/sbu222 25646526 PMC4332963

[77] Lee J, Zaki J, Harvey P, Ochsner K, Green MF, Zaki J., et al Schizophrenia patients are impaired in empathic accuracy. Psychol Med 2011;41(11):2297–304. 10.1017/S0033291711000614 21524334 PMC3928128

[78] Park S, Matthews N, Gibson C. Imitation, simulation, and schizophrenia. Schizophr Bull 2008;34(4):698–707. 10.1093/schbul/sbn048 18499703 PMC2632442

[79] Matthews N, Gold BJ, Sekuler R, Park S. Gesture imitation in schizophrenia. Schizophr Bull 2013;39(1):94–101. 10.1093/schbul/sbr062 21765171 PMC3523902

[80] Roder V, Mueller DR, Mueser KT, Brenner HD. Integrated psychological therapy (IPT) for schizophrenia: Is it effective? Schizophr Bull 2006;32(suppl 1):S81–93. 16916888 10.1093/schbul/sbl021 PMC2632544

[81] Kuipers E, Garety P, Fowler D, Freeman D, Dunn G, Bebbington P. Cognitive, emotional, and social processes in psychosis: Refining cognitive behavioral therapy for persistent positive symptoms. Schizophr Bull 2006;32(suppl 1):S24–31. 16885206 10.1093/schbul/sbl014 PMC2632539

[82] Kupper Z, Hoffmann H. Course patterns of psychosocial functioning in schizophrenia patients attending a vocational rehabilitation program. Schizophr Bull 2000;26(3):681–98. 10993406 10.1093/oxfordjournals.schbul.a033486

[83] Hoffmann H, Kupper Z. Relationships between social competence, psychopathology and work performance and their predictive value for vocational rehabilitation of schizophrenic outpatients. Schizophr Res 1997;23(1):69–79. 9050130 10.1016/S0920-9964(96)00082-5

[84] Hoffmann H, Kupper Z, Kunz B. Predicting schizophrenic outpatients' behavior by symptomatology and social skills. J Nerv Ment Dis 1998;186(4):214–22. 9569889 10.1097/00005053-199804000-00003

[85] Gallese V. Embodied simulation: From neurons to phenomenal experience. Phenomenology and the Cognitive Sciences 2005;4(1):23–48.

[86] Gallese V. Intentional attunement: A neurophysiological perspective on social cognition and its disruption in autism. Brain Res 2006;1079(1):15–24. 16680812 10.1016/j.brainres.2006.01.054

